# *Bifidobacterium longum* subsp. *infantis* EVC001 Administration Is Associated with a Significant Reduction in the Incidence of Necrotizing Enterocolitis in Very Low Birth Weight Infants

**DOI:** 10.1016/j.jpeds.2021.12.070

**Published:** 2022-01-12

**Authors:** Joseph Tobias, Amy Olyaei, Bryan Laraway, Brian K. Jordan, Stephanie L. Dickinson, Lilian Golzarri-Arroyo, Elizabeth Fialkowski, Arthur Owora, Brian Scottoline

**Affiliations:** 1 Department of Surgery, Oregon Health & Science University, Portland, OR; 2 Division of Neonatology, Department of Pediatrics, Oregon Health & Science University, Portland, OR; 3 Oregon Clinical and Translational Research Institute, Oregon Health & Science University, Portland, OR; 4 School of Public Health, Indiana University, Bloomington, IN

## Abstract

**Objective:**

To assess the effects of *Bifidobacterium longum* subsp. *infantis* EVC001 (*B infantis* EVC001) administration on the incidence of necrotizing enterocolitis (NEC) in preterm infants in a single level IV neonatal intensive care unit (NICU).

**Study design:**

Nonconcurrent retrospective analysis of 2 cohorts of very low birth weight (VLBW) infants not exposed and exposed to *B infantis* EVC001 probiotic at Oregon Health & Science University from 2014 to 2020. Outcomes included NEC incidence and NEC-associated mortality, including subgroup analysis of extremely low birth weight (ELBW) infants. Log-binomial regression models were used to compare the incidence and risk of NEC-associated outcomes between the unexposed and exposed cohorts.

**Results:**

The cumulative incidence of NEC diagnoses decreased from 11.0% (n = 301) in the no EVC001 (unexposed) cohort to 2.7% (n = 182) in the EVC001 (exposed) cohort (*P* < .01). The EVC001 cohort had a 73% risk reduction of NEC compared with the no EVC001 cohort (adjusted risk ratio, 0.27; 95% CI, 0.094–0.614; *P* < .01) resulting in an adjusted number needed to treat of 13 (95% CI, 10.0–23.5) for *B infantis* EVC001. NEC-associated mortality decreased from 2.7% in the no EVC001 cohort to 0% in the EVC001 cohort (*P* = .03). There were similar reductions in NEC incidence and risk for ELBW infants (19.2% vs 5.3% [*P* < .01]; adjusted risk ratio, 0.28; 95% CI, 0.085–0.698 [*P* = .02]) and mortality (5.6% vs 0%; *P* < .05) in the 2 cohorts.

**Conclusions:**

In this observational study of 483 VLBW infants, *B infantis* EVC001 administration was associated with significant reductions in the risk of NEC and NEC-related mortality. *B infantis* EVC001 supplementation may be considered safe and effective for reducing morbidity and mortality in the NICU.

Necrotizing enterocolitis (NEC) disproportionately affects preterm, very low birth weight (VLBW) infants (birth weight <1500 g).^1^ In the US, NEC has an incidence of 5%-10% among VLBW infants and carries an overall mortality of 23.5%, although mortality is as high as 50% among extremely low birth weight (ELBW) patients requiring surgery.^2^

The development of NEC has been strongly linked to dysbiosis of the preterm gut,^3^ which is thought to promote uncontrolled inflammation of an immunologically immature, intrinsically hyperreactive intestinal epithelium, culminating in epithelial and transmural necrosis.^4,5^ In support of this theory, proliferation of Proteobacteria has been shown to accentuate enteric inflammation and, in some cases, to precede NEC.^4,5–7^ Moreover, signaling of the intestinal immune receptor for lipopolysaccharide (ie, Toll-like receptor 4) by gram-negative bacteria is sufficient for the development of NEC in animal models, and, conversely, NEC cannot be induced in germfree animals.^5,6^

Changing the composition of the intestinal microbiota through enteral probiotic administration may promote a microbial community that attenuates or even prevents dysbiotic NEC. Meta-analyses of studies of probiotic administration in >10 000 patients have indicated with varying degrees of certainty that probiotics can reduce the incidence (or rate) of NEC and associated mortality in preterm infants.^7–9^ The generalizability of these results is limited by negative trials and the high degree of heterogeneity in probiotic formulation. As a result, it remains controversial whether to use probiotics to prevent NEC due to dysbiosis, as well as the strain, formulation, timing and dosing to use.

*Bifidobacterium longum* subspecies *infantis* (*B infantis*) is a mutualist colonizer of the human infant gut worldwide.^10,11^ The *B infantis* EVC001 strain encodes the complete gene cluster needed to metabolize the full range of prebiotic human milk oligosaccharides (HMOs), complex sugars in human milk that are otherwise indigestible by the infant.^12,13^ In turn, *B infantis* EVC001 functions as a natural symbiont in the infant gut, conferring such ecosystem services as colonization resistance to pathogens and bioactive metabolite production.^14–16^

Supplementing *B infantis* EVC001 to human milk-fed preterm infants in a neonatal intensive care unit (NICU) is safe and well-tolerated, colonizes the intestinal microbiota, lowers the abundance of NEC-associated pathogenic bacteria and fecal antibiotic resistance genes, and decreases enteric inflammation as measured by fecal calprotectin and cytokine levels.^17^ Notwithstanding these promising strain-specific findings, *B infantis* EVC001 as a single-strain probiotic has yet to be associated with reducing NEC incidence in preterm infants. Accordingly, the objective of this study was to evaluate the impact of *B infantis* EVC001 administration on the rate of NEC in at-risk preterm infants.

## Methods

A nonconcurrent retrospective cohort design was used to compare clinical outcomes in VLBW infants who did not receive *B infantis* EVC001 and VLBW infants administered *B infantis* EVC001 (Evivo; Evolve BioSystems). Study approval was granted by the Oregon Health & Science University (OHSU) Institutional Review Board (IRB 20336).

### Data Source

Data were collected from electronic medical record review of infants admitted to the OHSU Doernbecher Children’s Hospital Level IV NICU between January 2014 and November 2020. Data were identified using the OHSU Epic Research Data Warehouse and correlated with OHSU Vermont Oxford Network data until complete correlation was achieved. A minimum of 2 reviewers independently validated collected data.

### Eligibility Criteria

Eligible infants weighed <1500 g at birth; received full resuscitation and survived until day of life 3 (the earliest time at which VLBWs would have received at least 1 dose of EVC001); were fed a human milk–based diet consisting of mother’s milk, donor milk, or a combination thereof; were fed according to institutional guidelines incorporating best practices for NEC prevention, including a human milk–based diet, an initial period of trophic feeding, and gradual feeding advancement; and did not have hemodynamically significant congenital heart disease (CHD) (excluding hemodynamically significant ductus arteriosus), because CHD is an additional risk factor for NEC.^18^ Hemodynamically significant CHD was defined as lesions that have been independently associated with an increased risk for NEC (eg, hypoplastic left heart syndrome, interrupted aortic arch, coarctation of the aorta, truncus arteriosus, atrioventricular canal, atrioventricular canal–like ventricular septal defect, transposition of the great arteries, unobstructed tetralogy of Fallot).^18^ Infants with forms of CHD that did not include lesions independently associated with increased NEC risk were included in both cohorts.

### Exclusion Criteria

Excluded infants were those who underwent palliative delivery or unsuccessful resuscitation, died before day of life 4, were fed a non–human milk–based diet before 34 weeks postmenstrual age (PMA), had immunodeficiency or hemodynamically significant CHD as defined above, or, if in the EVC001 cohort, received fewer than 2 doses of EVC001.

### Cohorts

**Figure 1.**
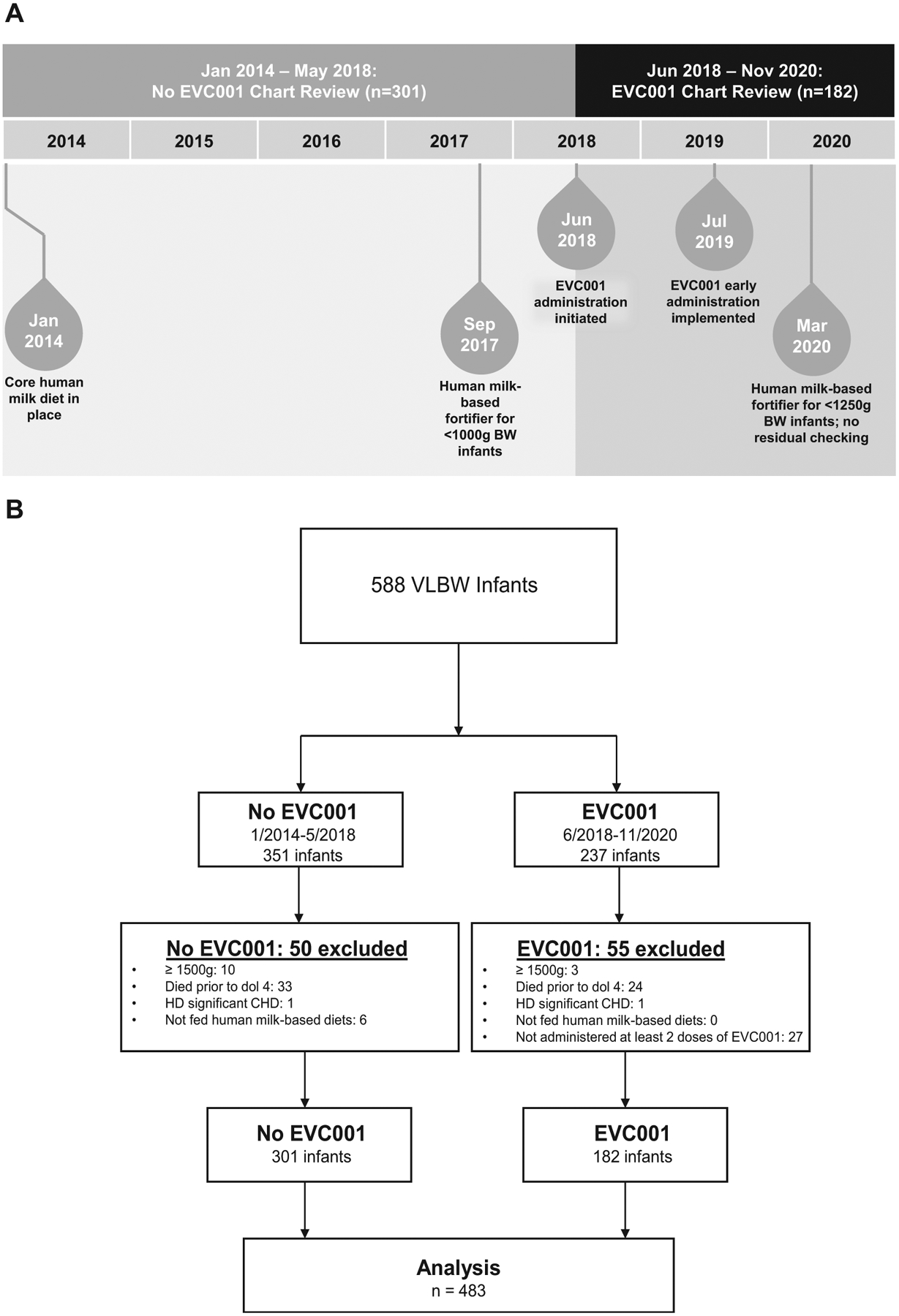
**A**, Study design including changes to standard of care during the chart review period. **B**, STROBE flow diagram identifying infants included in the analysis.

The reference, unexposed cohort (no EVC001) comprised VLBW infants admitted to the NICU between January 2014 and May 2018 who were not supplemented with *B infantis* EVC001 (Figure 1, A). The exposed cohort (EVC001) comprised VLBW infants admitted to the NICU between June 2018 and November 2020 who received at least 2 doses of *B infantis* EVC001. Inclusion criteria for the EVC001 cohort required that infants receive 2 or more doses of *B infantis* EVC001 to increase the probability of intestinal colonization.

### Core Human Milk–Based Diet

Each cohort was fed a human milk–based diet of mother’s milk, donor milk, or both. Feeding regimens consisted of an initial period of trophic feeding, followed by daily advancements as tolerated to a goal feeding volume of 150–160 mL/kg/day. Donor milk feeding was continued until at least 34 weeks PMA or for a minimum of 5 days in infants born at >34 weeks, after which donor milk was replaced with bovine milk–based formula if mother’s milk was unavailable. A bovine milk–based human milk fortifier (Similac HMF; Abbott) was used to meet the nutrient and energy needs of VLBW infants until September 2017; thereafter, human milk-based fortification (Prolacta, Prolacta) was used for ELBW infants. The use of bovine milk–based fortification was continued for infants weighing >1000 g but <1500 g at birth. As of March 2020, human milk–based fortification was expanded to all infants with birth weight <1250 g.

### *B infantis* EVC001

Infants in the EVC001 cohort received 8 billion colony-forming units of activated *B infantis* EVC001 suspended in 0.5 mL of medium-chain triglyceride oil daily via gastric tube before a morning feed. The product was produced in a dedicated production facility as a Food for Special Dietary Useunder US Food and Drug Administration guidelines. Rigorous food safety analyses, including pathogen testing and heavy metal analysis, were performed by an independent third-party laboratory. The strain identity of each lot was confirmed by whole genome sequencing and a shelf-life testing program ensured that the product was guaranteed to contain the minimum label claim of 8 billion colony-forming units per dose at the end of the shelf life. From June 2018 to July 2019, *B infantis* EVC001 administration was initiated at feeding volumes of 80–100 mL/kg/day. In August 2019, the administration protocol was revised to begin on the second day of trophic feeding. EVC001 administration was continued until 34 weeks PMA or for a minimum of 2 weeks, whichever was longer.

### Covariates

Variables examined as potential predictors, confounders, and effect modifiers were birth weight, sex, gestational age at birth, small for gestational age (SGA) status, the presence of non-hemodynamically significant CHD, antenatal steroid administration before delivery, defined as 1 or more maternal doses of betamethasone within 2 weeks of delivery, mode of delivery, and packed red blood cell transfusion within 72 hours of NEC diagnosis. The primary outcome was NEC Bell stage 2 or higher as abstracted from electronic medical record review and near real-time case tracking by at least 2 neonatologists.

### Primary Outcome Measure

The diagnosis of NEC was determined using the modified Bell staging system.^19^ NEC was assigned if the infant’s condition met the criteria for modified Bell stage 2 or greater. Cases of spontaneous intestinal perforation were excluded. Spontaneous intestinal perforation was defined as gastrointestinal perforation without signs of NEC. The diagnosis of NEC was confirmed by an independent review of each case by at least 2 neonatologists and at least 1 pediatric surgeon.

### Secondary Outcome Measures

Secondary outcome measures were NEC necessitating antibiotic therapy and supportive care (medical NEC), NEC necessitating operative intervention (surgical NEC), NEC-associated mortality (ie, deaths directly attributable to NEC), and day of life at diagnosis of NEC. Modified Bell staging was determined by electronic medical record review and real-time case tracking, as described above.

### Statistical Analyses

The Wilcoxon rank-sum test and Pearson χ^2^ or Fisher exact test were used to determine differences in demographic and clinical characteristics between exposed and unexposed cohorts for continuous and discrete variables, respectively. Log-binomial regression models were used to compare risks of NEC-associated outcomes between exposed and unexposed cohorts, accounting for potential effect modifiers and confounding by covariates. Moderation effects were evaluated by examining whether the magnitude of the exposure–NEC outcomes differed across subgroups of other covariates (ie, statistical significance of pairwise interaction terms). Potential confounders were first identified based on a *P* value <.20 in univariate models: sex, birth weight, gestational age at birth, and mode of delivery. The potential confounders were then included in models alongside exposure to determine whether regression coefficients were changed by ≥10%; however, no variables met that criterion. Risk ratios (RRs) and adjusted number needed to treat (NNT) were calculated as measures of association and exposure effect. A subgroup analysis of effects of Prolacta in the no EVC001 cohort was performed in infants weighing <1000 g at birth. In addition, NEC incidence was analyzed between cohorts separately in infants with birth weight <1000 g and ≥1000 g.

Overall NEC incidence and NEC risk with 95% CI were calculated for exposed and unexposed cohorts of VLBW infants of all birth weights and for a subgroup of ELBW infants. In both cohorts, the incidences of NEC-associated mortality, medical NEC, and surgical NEC were compared using the Fisher exact test. The 95% CIs were calculated using a normal approximation, with a continuity correction applied for data with few outcomes.

The relative risk of NEC by cohort was estimated with a log-binomial regression model adjusting for sex, birth weight, gestational age at birth, and mode of delivery. An adjusted NNT was calculated from the cohort coefficient of the model. The same model and calculations were applied to a subgroup of ELBW infants.

Statistical significance was assessed at an a value of 0.05 in our final models. All statistical analyses were performed using R version 3.6.3 with the tidyverse, ggpubr, psych, kableExtra, car, e1071, epitools, emmeans, meta, cmprsk, and survival packages (R Foundation for Statistical Computing) by collaborating biostatisticians at Indiana University School of Public Health.

## Results

Of the 588 VLBW infants identified during the study period, 105 infants were palliatively delivered, unsuccessfully resuscitated, died within the first 3 days of life, or were not fed a human milk–based diet (Figure 1, B). The remaining 483 infants who met our inclusion criteria consisted of 301 infants who were not exposed to *B infantis* EVC001 (no EVC001 cohort) and 182 infants who were exposed to the probiotic (EVC001 cohort). There were no significant differences in the measured covariates between the 2 cohorts except for sex and antenatal steroid administration (Table I). The mean gestational age at birth for both cohorts was 28 weeks, with a mean birth weight of 1045.4 g for the no EVC001 cohort and 1048.0 g for the EVC001 cohort. Most infants were delivered by cesarean. There were statistically significantly lower percentages of antenatal steroid administration (*P* = .03) and female infants (*P* = .04) in the EVC001 cohort. There were no significant between-group differences in any of the measured covariates in the subgroup analysis of ELBW infants.

**Figure 2.**
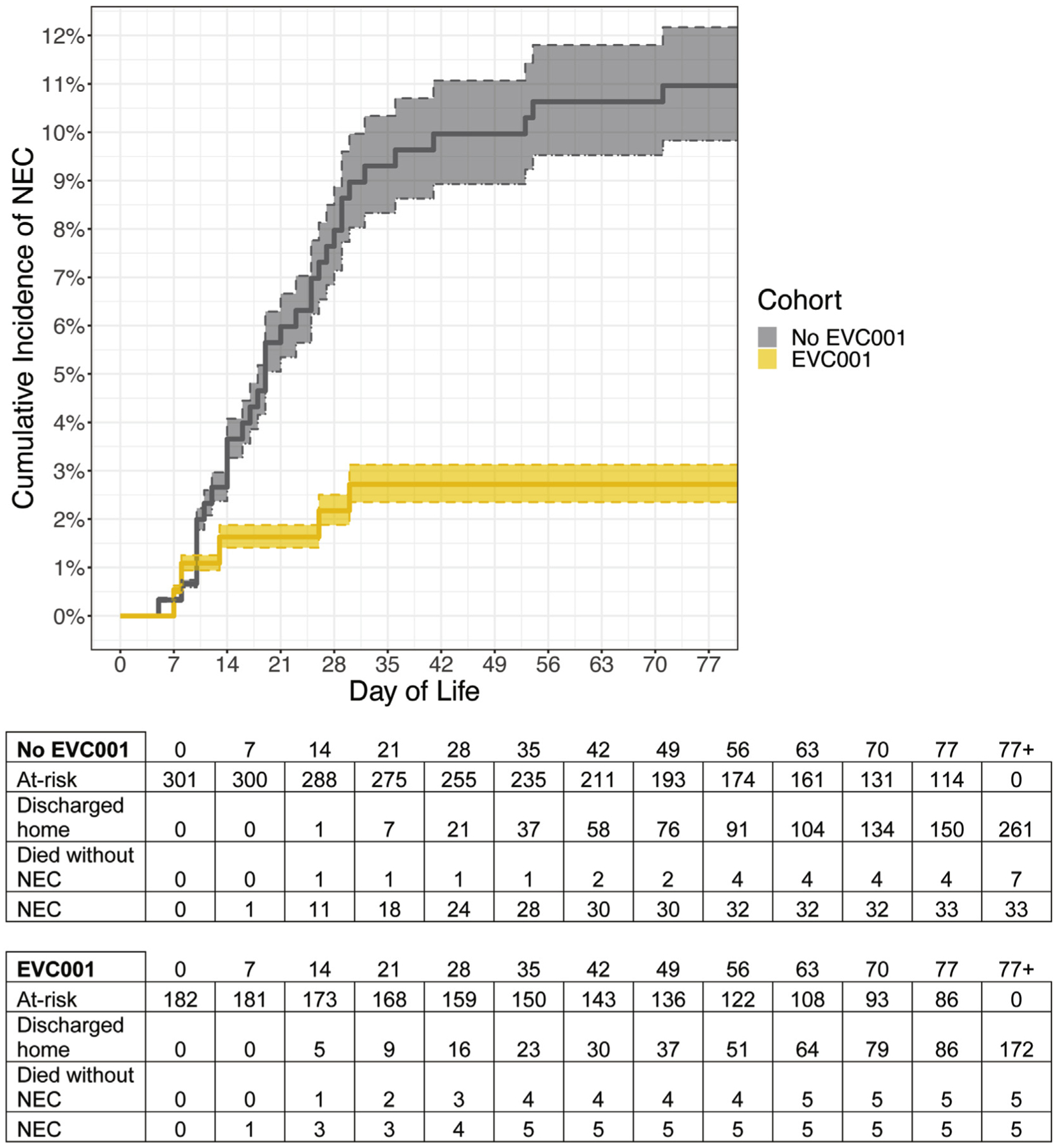
Cumulative incidence of NEC by day of life. Shaded regions denote 95% CIs around the estimates.

**Figure 3.**
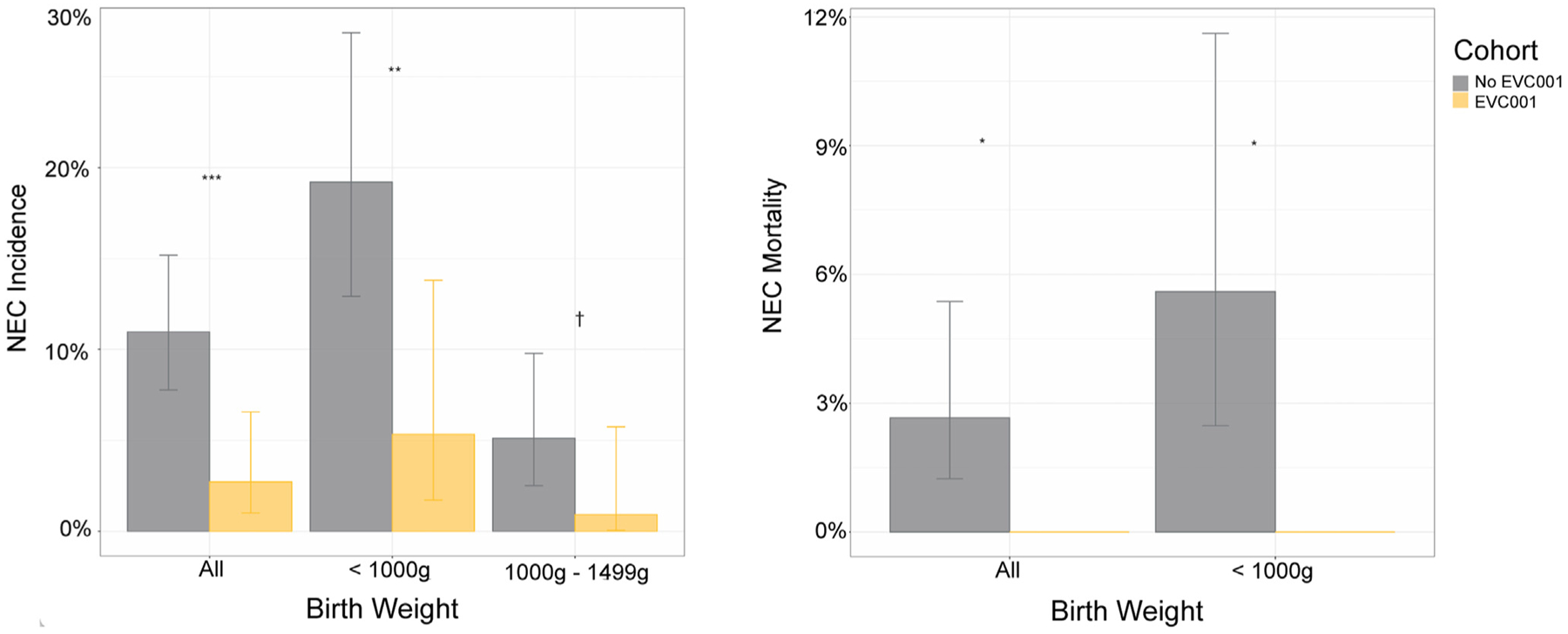
**A**, NEC incidence by birth weight and cohort. Error bars show 95% CIs around the estimates. ***P < .001; **P < .01; ^†^*P* < .1, Fisher exact test. **B**, NEC-related mortality rates by birth weight and cohort. Error bars show 95% CIs around the estimates. *P < .05, Fisher exact test.

Regarding the primary outcome, there were 33 cases of NEC (11.0%) in the no EVC001 cohort (Figure 2 [available at www.jpeds.com], Figure 3, A, and Table II), inclusive of the 9-month period after the introduction of human milk–based fortification in the ELBW population. No significant difference in the rate of NEC was observed between ELBW infants in the no EVC001 cohort during the 9 months of exclusive human milk use (19 of 95 ELBW infants [20.0%] before human milk–based fortification compared with 5 of 30 ELBW infants [16.7%] after human milk–based fortification; *P* = .80). Therefore, the no EVC001 cohort was analyzed as a single group for all outcomes, regardless of human milk fortification type. There were 5 cases of NEC (2.7%) in the EVC001 cohort. The difference in NEC rate between cohorts was statistically significant (*P* < .01). Log-binomial models demonstrated that infants in the no EVC001 cohort had a 73% higher cumulative incidence of NEC compared with infants in the EVC001 cohort (*P* < .01) after adjusting for differences in sex, birth weight, gestational age, and mode of delivery. The adjusted NNT based on these outcomes was 13. The association between EVC001 exposure and NEC incidence was modified by delivery mode (statistical interaction term: delivery mode × EVC cohort; *P* = .01) (Appendix; available at www.jpeds.com). For infants delivered by cesarean, the risk of NEC was 88% lower (RR, 0.12; 95% CI, 0.02–0.40) in the EVC001 cohort compared with the no EVC001 cohort. For infants delivered vaginally, the risk of NEC was not statistically significantly different between the 2 cohorts (RR, 1.8; 95% CI, 0.3–10.1), with vaginal deliveries representing approximately one-third of the total infants evaluated. Sex, birth weight, birth weight subgroup (ELBW), gestational age at birth, SGA status, and non-hemodynamically significant CHD were evaluated individually for interactions with treatment and were not found to be effect modifiers of NEC. The association between EVC001 exposure and NEC incidence was not modified by birth weight group (ELBW vs ≥1000 g; statistical interaction term: birth weight group × EVC001 cohort; *P* = .77).

Of note, there were 6 additional cases of NEC during the EVC001 epoch in VLBW infants without hemodynamically significant CHD who received no EVC001 (n = 5) or 1 dose of EVC001 (n = 1) before their diagnosis of NEC. In these cases, EVC001 was initiated when feeding volume reached 80–100 mL/kg/day, and the infants had not yet reached those volumes. Four of the 6 cases occurred prior to day of life 10, and the remaining 2 were prior to day of life 13.

Subgroup analysis was carried out to determine the effect of B *infantis* EVC001 administration on ELBW infants (birth weight <1000 g) and those with a birth weight of 1000–1499 g. ELBW infants demonstrated a statistically significant difference in NEC incidence between cohorts (*P* < .01), with an adjusted RR of 0.28 (95% CI, 0.09–0.70; *P* = .02) in ELBW infants (Figure 3, A and Table II). The adjusted NNT was 8. Notably, ELBW infants in the no EVC001 cohort received bovine milk–based fortification until the final 9 months of that cohort period (n = 30), whereas ELBW infants in the EVC001 cohort received human milk–based fortification. Although not statistically significant (*P* = .10), the incidence of NEC in infants with a birth weight of 1000–1499 g was higher in the no EVC001 cohort (9 of 176; 5.1%) compared with the EVC001 cohort (1 of 107; 0.9%), with an adjusted RR of 0.19 (95% CI, 0.01–0.97).

**Figure 4.**
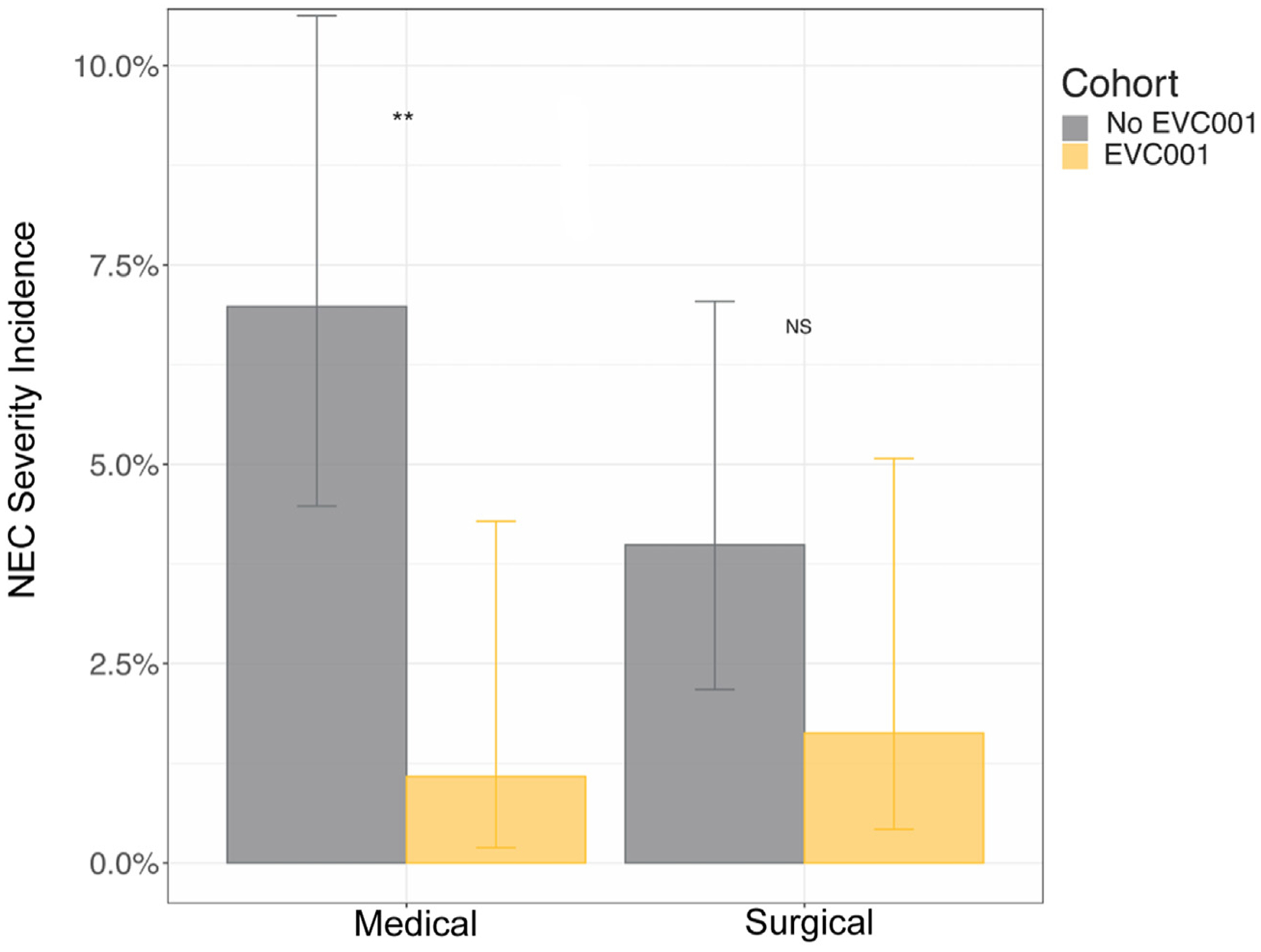
NEC incidence by severity (medical and surgical). Error bars show 95% CIs around estimates. **P < .01; NS, *P* > .1, Fisher exact test.

There was a statistically significant difference in the incidence of medical NEC between the no EVC001 and EVC001 cohorts (*P* < .01), but not in surgical NEC (*P* = .18) (Figure 4 [available at www.jpeds.com] and Table II). Among infants who developed NEC, there was no statistically significant difference in the incidence of medical vs surgical NEC (*P* = .37).

The NEC-associated mortality rate was 2.7% of all infants in the no EVC001 cohort (Table II and Figure 3, B) and 0% in the EVC001 cohort. The difference in NEC-associated mortality between cohorts was statistically significant (*P* = .03). There also was a statistically significant difference in the ELBW subgroup analysis of NEC-associated mortality (*P* < .05). Among infants diagnosed with NEC, the mortality rate was 24.2% (8 of 33) in the no EVC001 cohort, compared with 0% (0 of 5) in the EVC001 cohort (*P* = .56). The overall mortality was 6.6% in the no EVC001 cohort (4.0% non-NEC mortality) and 2.7% (2.7% non-NEC mortality) in the EVC001 cohort (*P* = .06).

No adverse effects directly attributable to the probiotic were observed with *B infantis* EVC001 administration, including no cases of *B infantis* bacteremia or other infections related to *B infantis* (>4500 patient-days). Nine infants in the no EVC001 cohort and 1 infant in the EVC001 cohort developed late-onset infection (*P* = .13). The late-onset infections in the no EVC001 cohort included 5 cases of bacteremia/culture-positive sepsis, 3 urinary tract infections, and 1 case of ventilator-associated pneumonia. In comparison, there was 1 case of bacteremia/culture-positive sepsis in the EV001 cohort.

## Discussion

In this retrospective cohort study of 483 VLBW infants in a single-center level IV NICU over nearly 7 years, we observed a significant reduction in NEC among VLBW human milk–fed infants associated with daily administration of the infant gut symbiont *B longum* subsp. *infantis* (*B infantis*) EVC001. The cumulative incidence of NEC in those infants given more than 1 dose of *B infantis* EVC001 decreased from 11% to 2.7%, a 73% risk reduction. There were no cases of NEC-associated mortality in the EVC001 cohort, which was significant for both the overall EVC001 cohort and the ELBW subgroup. In comparison, 24.2% of infants who developed NEC in the no EVC001 cohort died. Importantly, no adverse effects were attributed to *B infantis* EVC001 administration, including no cases of *B infantis* bacteremia or sepsis. In light of the 2021 statement from the American Academy of Pediatrics Committee on the Fetus and Newborn, this study and the reported findings support the recommendation that “centers choosing to administer probiotics should carefully document outcomes, adverse events, and safety.^20^

Care improvement measures were undertaken during the study period, several of which were aimed at optimizing nutrition and NEC reduction. Most relevant was the use of an exclusive human milk diet for ELBW infants, followed by expansion to infants of birth weight <1250 g. Although there were no differences between subgroups of VLBWs with respect to the mode of delivery, birth weight, gestational age at birth, SGA status, and the presence of CHD, there were marginally significant differences in the rate of antenatal steroid administration and sex distribution. Given that antenatal steroids reduce the risk of NEC in preterm infants,^21^ a higher rate of antenatal steroid use in the no EVC001 cohort would have been more likely to mitigate the observed benefit of *B infantis* EVC001 administration. The lower proportion of females in the EVC001 cohort would have similarly mitigated the observed benefit of EVC001 administration.^22^ Taken together, our findings support the safe use of *B infantis* EVC001 as a probiotic to reduce the incidence of NEC in the most at-risk NICU patients.

There is mechanistic evidence for the efficacy of *B infantis EVC001* in NEC reduction.^23^ *B infantis* is a foundational colonizer of the human milk–fed infant gut that appears to have co-evolved with humans to metabolize HMOs optimally.^24,25^ *B infantis* EVC001 harbors genes that facilitate the intracellular transport and complete metabolism of the full array of HMOs,^13^ properties that have thus far not been identified in most other strains. Oligosaccharide transporters and glycosidases confer a growth advantage relative to other microoragnisms, enabling *B infantis* EVC001 to predominate within the human milk–fed infant gut.

*B infantis* EVC001 colonization is hypothesized to provide important ecosystem services.^15^ *B infantis* EVC001 confers colonization resistance to pathogens by competitive growth and by fermenting HMOs into organic acids, such as lactate and acetate, that reduce intestinal pH and inhibit the growth of pathogenic species, including *Enterobacteriaceae* and *Clostridia*.^26^ Fermentation products also strengthen intestinal barrier function and exert anti-inflammatory effects.^17,27,28^ In particular, indole-containing tryptophan metabolites, enriched in the stools of human milk–fed infants supplemented with *B infantis* EVC001, have been shown to down-regulate Toll-like receptor 4 signaling via the aryl hydrocarbon receptor pathway.^29–31^

Human milk feeding in concert with this evolutionarily co-evolved bacterium may influence the intestinal microbiota through colonization resistance, modulation of the intestinal inflammatory environment and the host immune system. Consistent with this concept, *B infantis* EVC001 has been shown to reduce the burden of antibiotic resistance genes in the stool of human milk–fed term and preterm infants,^17,32^ reduce fecal markers of intestinal inflammation,^17^ and modulate systemic inflammation,^23^ possibly in a sustained manner.

Of 56 trials included in the most recent Cochrane meta-analysis (2020) of probiotics to prevent NEC, 14 trials used single-strain formulations containing other *Bifidobacteria* species, but none used *B infantis*.^8^ Trials that included *B infantis* did so only in multistrain formulations.^8^ Similarly, of 63 trials analyzed in a 2020 American Gastroenterological Association network meta-analysis (spanning research from 1986 to 2019), 15 trials used *B infantis* in multistrain formulations, most often combined with *Lactobacillus*, and no studies used *B infantis* as a single-strain probiotic.^9^ Notably, the most effective multistrain formulations in both meta-analyses contained *B infantis*.

This report adds to the diverse body of evidence supporting probiotic use as an effective means of NEC reduction while emphasizing the challenge to neonatology to provide improved clarity regarding, but not limited to, optimal strain(s), formulation, dosing, duration, target population, and dietary substrate—in particular, an exclusive human milk diet. This challenge includes mechanistic research to provide the biological basis for the reported clinical effects, an area that has been underresourced in proportion to the impact of NEC. Even less well understood are the long-term effects of microbiome alteration on the developing gut and overall health. It remains challenging to interpret the large quantity of available evidence to select a single-strain or multistrain probiotic formulation to prevent NEC in preterm infants. Based on outcomes observed in this study, and in light of its unique symbiotic, genetic, ecological, and biochemical properties, *B infantis* EVC001 is a promising candidate. More broadly, the results of this study suggest that a given probiotic formulation must have established efficacy and a viable mechanism of action in the infant gut.

The results of this study are limited by its single-center, retrospective, observational design, as well as by an absence of fecal sampling to confirm that *B infantis* EVC001 administration led to successful modulation of the preterm intestinal microbiota. Thus, the observed reduction in NEC incidence and risk associated with *B infantis* EVC001 is restricted to an association and is not necessarily causal. In addition, reporting the NNT here is a valuable measure for clinicians but has limitations. For example, changes in concomitant treatments over time may make the NNT somewhat unreliable in comparing pre-implementation and post-implementation cohorts. During the nearly 7-year chart review period, there were evolutions in patient care and unmeasured confounders that may have influenced the difference in NEC rates, including the addition of human milk–based fortifier for ELBW infants during the late no EVC001 period, followed by the addition to infants with birth weight 1000–1249 g late in the EVC001 exposure period. Although the addition of an exclusive human milk diet for ELBW infants did not result in a detectable decrease in NEC in the no EVC001 epoch, the time period was insufficient for detecting a potential effect on NEC reduction in this population. Nonetheless, the combination of an exclusive human milk diet and *B infantis* EVC001 yielded an apparent reduction in NEC that remains important. Other care changes implemented during the period applied to both epochs and did not apply to care thought to affect NEC. Finally, although there was an absence of fecal colonization data from EVC001 recipients, this has been welldemonstrated in recent publications.^14,17^

In this retrospective electronic medical record review, administration of *B longum* subsp. *infantis* EVC001 as a single strain to VLBW infants was associated with a significant decrease in NEC and NEC-associated mortality, including ELBW infants. The effect in ELBW infants was observed in combination with an exclusive human milk diet. Probiotic administration in this study was part of a comprehensive NEC prevention strategy implemented for all infants of birth weight <1500 g. Based on these findings, *B infantis* EVC001 administration can be considered a safe and effective method for preventing NEC in the NICU and modifying the dysbiosis thought to underpin a significant proportion of NEC.

## Supplementary Material

Appendix

Supplementary data

## Figures and Tables

**Table I.** Demographics

Characteristics	All VLBWs			ELBWs (<1000 g)		
	No EVC001 (N = 301)	EVC001 (N = 182)	*P* value	No EVC001 (N = 125)	EVC001 (N = 75)	*P* value
Female sex, %	52.2	42.3	.04*	53.6	44.0	.19*
Birth weight, g, mean (range)	1045.4 (325–1490)	1048.0 (358–1498)	.97*	758.1 (325–992)	779.1 (358–999)	.38*
Gestational age at birth, wk, mean (range)	28.3 (23.3–35.1)	28.4 (23.6–34.9)	.46*	26.0 (23.3–31.7)	26.6 (23.6–34.1)	.17*
SGA status, %	15.9	14.8	.74^†^	19.2	16.0	.57^†^
Cesarean delivery, %	77.1	74.2	.47^†^	80.0	76.0	.51^†^
CHD, %^‡^	4.0	7.1	.13^†^	3.2	5.3	.71^†^
Antenatal steroid use, %	90.4	83.5	.03^†^	90.4	85.3	.28^†^

* By the Wilcoxon rank-sum test.

† By the Pearson χ^2^ test.

‡ Nonhemodynamically significant.

**Table II.** NEC outcomes

Outcomes	All VLBWs			ELBWs (<1000 g)		
	No EVC001 (N = 301)	EVC001 (N = 182)	*P* value	No EVC001 (N = 125)	EVC001 (N = 75)	*P* value
NEC, %	11.0	2.7	<.01*	19.2	5.3	<.01*
RR (95% CI)	0.27 (0.09–0.61)		<.01^†^	0.28 (0.09–0.70)		.02^†^
NNT (95% CI)	13 (10.0–23.5)			8 (5.7–17.3)		
NEC-related mortality, %	2.7	0	.03*	5.6	0	<.05*
NEC onset, day of life, mean	23.5	16.8	.30^‡^	24.0	19.3	.65^‡^
Medical NEC (Bell stage II), %	7.0	1.1	<.01*	12.0	2.7	.03*
Surgical NEC (Bell stage III), %	4.0	1.6	.18*	7.2	2.7	.21*

* By the Fisher exact test.

† By log binomial regression adjusted for sex, birth weight, gestational age at birth, and mode of delivery.

‡ By the Wilcoxon rank-sum test.

## Data Availability

Data sharing statement available at www.jpeds.com.

## Abbreviations

CHD: Congenital heart disease
ELBW: Extremely low birth weight
HMO: Human milk oligosaccharide
NEC: Necrotizing enterocolitis
NICU: Neonatal intensive care unit
NNT: Number needed to treat
OHSU: Oregon Health & Science University
PMA: Postmenstrual age
RR: Risk ratio
SGA: Small for gestational age
VLBW: Very low birth weight
