# Supplementary figures and images for: Bifidobacterium longum subsp. infantis EVC001 Administration Is Associated with a Significant Reduction in the Incidence of Necrotizing Enterocolitis in Very Low Birth Weight Infants
Source: J Pediatr. Author manuscript; Available in PMC 2023 Jun 23. (PMC10289059; doi:10.1016/j.jpeds.2021.12.070)

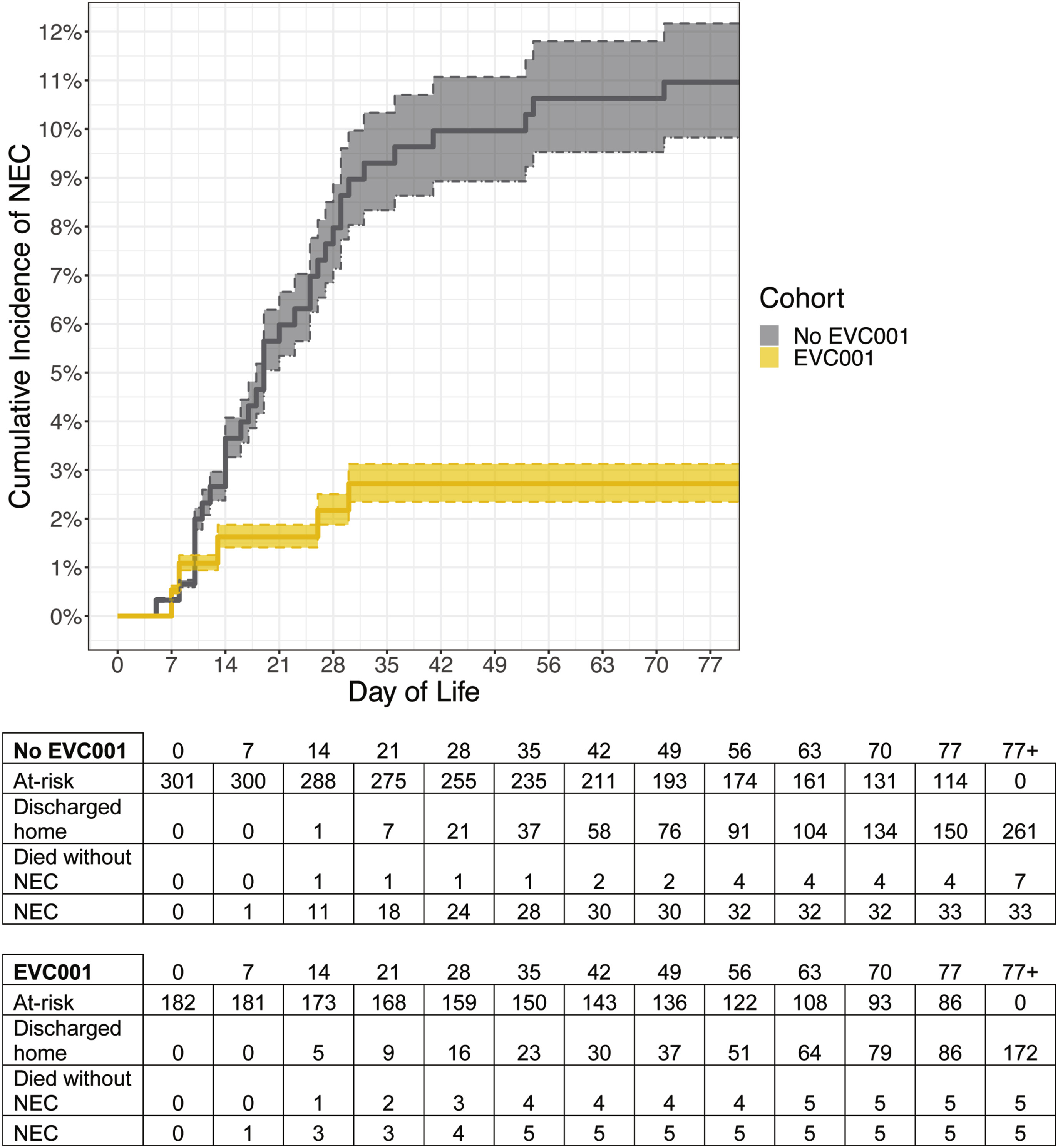

Supplement: Appendix [file NIHMS1906110-supplement-Appendix.jpg]
