# Supplementary material for: Bifidobacterium longum subsp. infantis EVC001 Administration Is Associated with a Significant Reduction in the Incidence of Necrotizing Enterocolitis in Very Low Birth Weight Infants
Source: J Pediatr. Author manuscript; Available in PMC 2023 Jun 23. (PMC10289059; doi:10.1016/j.jpeds.2021.12.070)
Supplement: Supplementary data [file NIHMS1906110-supplement-Supplementary_data.pdf]

Code

Appendix 1

07/15/2021

Code

Code

Code

Log Binomial regression (adjusted) with effect modifier

Code

```
##
## Call:
## glm(formula = NEC01 ~ Cohort + Sex + BW + Weeks + Cohort * `Mode of del`,
##     family = binomial(link = "log"), data = df)
##
## Deviance Residuals:
##     Min       1Q   Median       3Q      Max
## -1.0036  -0.4523  -0.2905  -0.1672   2.9490
##
## Coefficients:
##                               Estimate Std. Error z value Pr(>|z|)
## (Intercept)                  0.7663929  2.0450308   0.375  0.70784
## CohortEvivo                 -2.1136367  0.7200834  -2.935  0.00333 **
## SexM                        -0.3884195  0.3237545  -1.200  0.23024
## BW                          -0.0011494  0.0008039  -1.430  0.15277
## Weeks                       -0.0537502  0.0914723  -0.588  0.55679
## `Mode of del`VD             -1.5011510  0.7187669  -2.089  0.03675 *
## CohortEvivo:`Mode of del`VD  2.8644492  1.1448699   2.502  0.01235 *
## ---
## Signif. codes:  0 '***' 0.001 '**' 0.01 '*' 0.05 '.' 0.1 ' ' 1
##
## (Dispersion parameter for binomial family taken to be 1)
##
##     Null deviance: 266.15  on 482  degrees of freedom
## Residual deviance: 232.55  on 476  degrees of freedom
##   (4 observations deleted due to missingness)
## AIC: 246.55
##
## Number of Fisher Scoring iterations: 7
```

Relative Risk:

Code

```
##                (Intercept)                 CohortEvivo
##                      2.152                       0.121
##                       SexM                          BW
##                      0.678                       0.999
##                      Weeks            `Mode of del`VD
##                      0.948                       0.223
## CohortEvivo:`Mode of del`VD
##                     17.539
```

95% confidence intervals:

Code

```
##                              2.5 %  97.5 %
## (Intercept)                  0.035 164.443
## CohortEvivo                  0.020   0.389
## SexM                         0.349   1.275
## BW                           0.997   1.000
## Weeks                        0.779   1.138
## `Mode of del`VD              0.036   0.723
## CohortEvivo:`Mode of del`VD 2.052 218.422
```

Effect modifiers - Weight Subgroups

```
##                                  Estimate Std. Error z value Pr(>|z|)
## (Intercept)                        -1.371      0.210  -6.546    0.000
## Cohort_subgroupControl VLBW        -1.519      0.482  -3.148    0.002
## Cohort_subgroupEvivo ELBW          -2.125      1.007  -2.111    0.035
## Cohort_subgroupEvivo VLBW         -17.015    898.793  -0.019    0.985
## SexM                               -0.743      0.412  -1.805    0.071
## Cohort_subgroupControl VLBW:SexM    0.565      0.773   0.732    0.464
## Cohort_subgroupEvivo ELBW:SexM      1.601      1.204   1.330    0.184
## Cohort_subgroupEvivo VLBW:SexM     14.986    898.793   0.017    0.987
```

Code


```
##                                  Estimate Std. Error z value Pr(>|z|)
## (Intercept)                        -1.371      0.210  -6.546    0.000
## Cohort_subgroupControl VLBW        -1.519      0.482  -3.148    0.002
## Cohort_subgroupEvivo ELBW          -2.125      1.007  -2.111    0.035
## Cohort_subgroupEvivo VLBW         -17.015    898.793  -0.019    0.985
## SexM                               -0.743      0.412  -1.805    0.071
## Cohort_subgroupControl VLBW:SexM    0.565      0.773   0.732    0.464
## Cohort_subgroupEvivo ELBW:SexM      1.601      1.204   1.330    0.184
## Cohort_subgroupEvivo VLBW:SexM     14.986    898.793   0.017    0.987
```


Code

```
##                                Estimate Std. Error z value Pr(>|z|)
## (Intercept)                      -2.176      0.963  -2.259    0.024
## Cohort_subgroupControl VLBW      -1.316      3.118  -0.422    0.673
## Cohort_subgroupEvivo ELBW        -0.333      2.731  -0.122    0.903
## Cohort_subgroupEvivo VLBW        -5.575      8.738  -0.638    0.523
## BW                                0.001      0.001   0.564    0.573
## Cohort_subgroupControl VLBW:BW    0.000      0.003  -0.103    0.918
## Cohort_subgroupEvivo ELBW:BW     -0.001      0.003  -0.353    0.724
## Cohort_subgroupEvivo VLBW:BW      0.002      0.007   0.258    0.797
```

Code

```
##                                   Estimate Std. Error z value Pr(>|z|)
## (Intercept)                         -5.535      2.384  -2.322    0.020
## Cohort_subgroupControl VLBW          1.388      5.129   0.271    0.787
## Cohort_subgroupEvivo ELBW            9.120      8.262   1.104    0.270
## Cohort_subgroupEvivo VLBW           11.710     17.641   0.664    0.507
## Weeks                                0.148      0.089   1.662    0.096
## Cohort_subgroupControl VLBW:Weeks   -0.109      0.175  -0.620    0.535
## Cohort_subgroupEvivo ELBW:Weeks     -0.398      0.321  -1.241    0.215
## Cohort_subgroupEvivo VLBW:Weeks     -0.520      0.617  -0.843    0.399
```

Code

Code

```
##                                        Estimate Std. Error z value Pr(>|z|)
## (Intercept)                              -1.725      0.214  -8.072    0.000
## Cohort_subgroupControl VLBW              -1.220      0.405  -3.011    0.003
## Cohort_subgroupEvivo ELBW                -1.032      0.529  -1.951    0.051
## Cohort_subgroupEvivo VLBW                -2.797      1.017  -2.750    0.006
## `SGA?`Yes                                 0.338      0.413   0.819    0.413
## Cohort_subgroupControl VLBW:`SGA?`Yes    -0.572      1.117  -0.512    0.609
## Cohort_subgroupEvivo ELBW:`SGA?`Yes     -15.968   1721.057  -0.009    0.993
## Cohort_subgroupEvivo VLBW:`SGA?`Yes     -14.203   1539.360  -0.009    0.993
```

Code

```
##                                              Estimate Std. Error z value
## (Intercept)                                    -1.470      0.183  -8.032
## Cohort_subgroupControl VLBW                    -1.334      0.388  -3.433
## Cohort_subgroupEvivo ELBW                      -1.880      0.718  -2.618
## Cohort_subgroupEvivo VLBW                     -17.917   1112.976  -0.016
## `Mode of del`VD                                -1.749      0.997  -1.755
## Cohort_subgroupControl VLBW:`Mode of del`VD     0.768      1.445   0.532
## Cohort_subgroupEvivo ELBW:`Mode of del`VD       2.902      1.386   2.094
## Cohort_subgroupEvivo VLBW:`Mode of del`VD      17.768   1112.977   0.016
##                                              Pr(>|z|)
## (Intercept)                                     0.000
## Cohort_subgroupControl VLBW                     0.001
## Cohort_subgroupEvivo ELBW                       0.009
## Cohort_subgroupEvivo VLBW                       0.987
## `Mode of del`VD                                 0.079
## Cohort_subgroupControl VLBW:`Mode of del`VD     0.595
## Cohort_subgroupEvivo ELBW:`Mode of del`VD       0.036
## Cohort_subgroupEvivo VLBW:`Mode of del`VD       0.987
```

Code

```
##                                     Estimate Std. Error z value Pr(>|z|)
## (Intercept)                           -1.618      0.183  -8.852    0.000
## Cohort_subgroupControl VLBW           -1.560      0.413  -3.781    0.000
## Cohort_subgroupEvivo ELBW             -1.259      0.519  -2.425    0.015
## Cohort_subgroupEvivo VLBW             -2.967      1.012  -2.933    0.003
## CHDYes                               -15.769   1808.042  -0.009    0.993
## Cohort_subgroupControl VLBW:CHDYes    17.560   1808.042   0.010    0.992
## Cohort_subgroupEvivo ELBW:CHDYes       1.259   2556.958   0.000    1.000
## Cohort_subgroupEvivo VLBW:CHDYes       2.967   2172.997   0.001    0.999
```

P-values from effect modifier model evaluating birth weight subgroup*cohort, with the baseline being ‘No EVC001 ELBW infants’. The ‘All infants’ column contains the resulting P-values of the covariate cohort interactions without subgroup analysis.

**Covariate**	No EVC001, ≥ 1000g BW subgroup (n=176)	EVC001, ELBW subgroup (n=75)	EVC001, ≥ 1000g BW subgroup (n=107)	All infants (n=483)
Sex	0.46	0.18	0.99	0.15
Birth weight	0.92	0.72	0.80	0.69
GA at birth	0.54	0.22	0.40	0.21
SGA	0.61	0.99	0.99	0.99
Mode of Delivery	0.60	0.04	0.99	0.01
CHD	0.99	1.00	1.00	0.99
